# Multigrain indexing of unknown multiphase materials

**DOI:** 10.1107/S1600576716003691

**Published:** 2016-03-24

**Authors:** Christian Wejdemann, Henning Friis Poulsen

**Affiliations:** a Department of Physics, Technical University of Denmark, Building 307, Fysikvej, Kongens Lyngby, 2800, Denmark

**Keywords:** indexing, multigrain crystallography, three-dimensional X-ray diffraction microscopy, 3DXRD

## Abstract

A multigrain indexing algorithm for use with samples comprising an arbitrary number of known or unknown phases is presented. No *a priori* crystallographic knowledge is required. The algorithm applies to data acquired with a monochromatic beam and a conventional two-dimensional detector for diffraction. Initially, candidate grains are found by searching for crystallographic planes, using a Dirac comb convoluted with a box function as a filter. Next, candidate grains are validated and the unit cell is optimized. The algorithm is validated by simulations. Simulations of 500 cementite grains and ∼100 reflections per grain resulted in 99.2% of all grains being indexed correctly and 99.5% of the reflections becoming associated with the right grain. Simulations with 200 grains associated with four mineral phases and 50–700 reflections per grain resulted in 99.9% of all grains being indexed correctly and 99.9% of the reflections becoming associated with the right grain. The main limitation is in terms of overlap of diffraction spots and computing time. Potential areas of use include three-dimensional grain mapping, structural solution and refinement studies of complex samples, and studies of dilute phases.

## Abstract 2

A multigrain indexing algorithm for use with samples comprising an arbitrary number of known or unknown phases is presented.

## 1. Introduction   

Multigrain crystallography is a relatively new approach (Lauridsen *et al.*, 2001 ▸; Poulsen, 2004 ▸; Sørensen, Schmidt *et al.*, 2012 ▸) whose aim is to provide a crystallographic description of each grain within a polycrystal or a powder specimen. The technique is complementary to traditional crystallographic analysis based on either single crystals or averaging over an ensemble of grains. The experimental setup is in the simplest case identical to that typically used in single-crystal X-ray crystallography, with a monochromatic beam, a fully illuminated sample in transmission geometry on a rotary table and a two-dimensional detector. The images acquired during a rotation of the sample may comprise up to a million diffraction spots from the grains simultaneously illuminated. A key step in the analysis of such data is a multigrain indexing program. Provided spot overlap is not excessive, programs have been developed that can index up to 3000 grains simultaneously (Lauridsen *et al.*, 2001 ▸; Wright, 2005 ▸; Ludwig *et al.*, 2009 ▸; Moscicki *et al.*, 2009 ▸; Bernier *et al.*, 2011 ▸; Sharma *et al.*, 2012 ▸; Schmidt, 2014 ▸). Once grains have been indexed, all the tools of single-crystal crystallography can be exploited for analysis of each of the grains. As examples, we mention reciprocal space mapping (Jakobsen *et al.*, 2007 ▸; Wejdemann *et al.*, 2013 ▸), defect studies (Ungár *et al.*, 2010 ▸), and in particular the solution and refinement of each grain (Schmidt *et al.*, 2003 ▸; Sørensen, Schmidt *et al.*, 2012 ▸). As illustrated, for example, in work on the compound [Cu(C_2_O_2_H_3_)_2_]·H_2_O, the refinement can be on a par with single-crystal results and clearly superior to results from state-of-the-art powder diffraction (Vaughan *et al.*, 2004 ▸).

Provided diffraction data are acquired with a high spatial resolution two-dimensional camera close to the sample, the multigrain indexing routines can also be used to generate three-dimensional maps of grains, their orientations and their stresses. This is enabled by tomographic type reconstruction algorithms, similar to classic computed tomography scanning but with diffraction contrast replacing absorption contrast. This has led to the establishment of the techniques known as three-dimensional X-ray diffraction microscopy (3DXRD; Poulsen, 2004 ▸; Hefferan *et al.*, 2009 ▸) and diffraction contrast tomography (Ludwig *et al.*, 2009 ▸; Reischig *et al.*, 2013 ▸). Typically the sample dimensions are ∼1 mm, while the spatial resolution is 1–3 µm. By stitching sub-volumes together, maps with up to 20 000 grains have been assembled. As examples of applications, see Offerman *et al.* (2002 ▸), Schmidt *et al.* (2008 ▸), King *et al.* (2008 ▸), Aydıner *et al.* (2009 ▸), Oddershede *et al.* (2012 ▸) and Hefferan *et al.* (2012 ▸).

A main limitation of the previous work has been that it applies almost exclusively to monophase materials. Furthermore, the indexing programs above all assume the space group and unit cell of the material to be known. One straightforward way to generalize the previous work is to apply the multigrain indexing and/or grain mapping algorithms repeatedly, once for each phase (Jimenez-Melero *et al.*, 2011 ▸; Sørensen, Hakim *et al.*, 2012 ▸), but this still requires the phases to be known. To our knowledge there have only been two proposals for dealing with unknown phases, both summarized by Sørensen, Schmidt *et al.* (2012 ▸). In the first study the diffraction data from five grains with different unit-cell parameters and orthorhombic or monoclinic symmetry were superposed, and a fast-Fourier-transform-based approach was used to index them. In the second study, 12 crystals of an ‘unknown’ monophase compound with a unit-cell volume of 2942 Å^3^ were successfully indexed by rotating two copies of the same data sets with respect to each other and searching for resonances (see also Schmidt, 2014 ▸).

Generally speaking, the unit cell of any structure is defined by three lattice vectors. Hence, a search and optimization procedure in the nine-dimensional space spanned by these three lattice vectors will provide an indexing of all grains. However, to our knowledge, such an approach is computationally not feasible.

In this article, we report a multigrain indexing routine that involves searching and optimization in a three-dimensional space. This is computationally feasible, and the work presented is in fact performed with MATLAB (The MathWorks Inc., Natick, MA, USA) code on a single-core PC. The algorithm can be applied to an arbitrary ensemble of grains belonging to an arbitrary number of known or unknown phases. Notably, the efficiency does not depend on the number of phases. The only limitations are in terms of overlap of diffraction spots and the signal-to-noise ratio of the reflections. We demonstrate the method using full-scale simulations.

## 2. Algorithm   

The indexing procedure below is based only on the position of the reflections, not their intensity. There are no *a priori* assumptions except for a minimum and maximum length of diffraction vectors given by parameters *q*_min_ and *q*_max_, respectively. For ease of presentation, in the following we assume an X-ray diffraction setup with a monochromatic beam, a rotary table and a far-field two-dimensional detector (implying that the sample dimensions are negligible or comparable to the detector pixel size). Furthermore, we assume the diffraction spots are already harvested and represented as diffraction vectors (reflections) in a coordinate system fixed to the sample.

**Figure 1:**
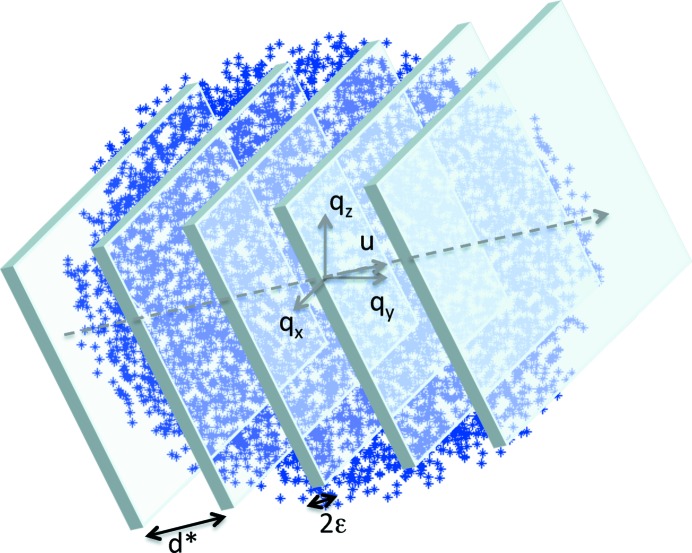
Illustration of the basic principle of the indexing algorithm. An optimization is performed with respect to sets of lattice planes, defined by a distance d* and a direction 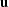, which comprise a maximum density of experimentally observed reflections.

The algorithm that we have developed essentially indexes grains independently. It is inspired by the *DIRAX* algorithm (Duisenberg, 1992 ▸) for indexing of single crystals in the presence of outliers. The concept is first to search for sets of equidistant lattice planes in the full set of reflections. Such sets are represented by the direction of the plane normal, 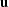, a unit vector, and by the distance between adjacent planes, d* (see Fig. 1 ▸). The number of experimentally determined reflections ‘lying on such planes’ can be counted. Candidate grains are defined by local maxima in the number of reflections on such planes as determined by a three-dimensional search in 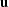 and d*. Candidate grains are associated with a subset of the reflections – those ‘lying on the planes’. This subset is refined by defining a new direction of the lattice plane normal and applying the above procedure to the subset already found.

In a second step a candidate grain is validated or rejected. If validated, a set of basis vectors in direct space is determined, and the reflections corresponding to the grain are identified within the full set of reflections. In a third step, the unit cell is optimized by techniques similar to those well known from conventional crystallography.

Once a grain has been identified the associated reflections are removed from the pool of all reflections. The entire procedure is then repeated. The indexing algorithm stops if the number of remaining (non-indexed) reflections becomes very low, or if no progress in terms of number of grains indexed has been reached within a certain number of iterations.

Once the grains have been indexed, multigrain studies can exploit the tools of single-crystal crystallography. This is outside the scope of this article; we refer the reader to the review by Sørensen, Schmidt *et al.* (2012 ▸).

The main steps of the algorithm are now described in detail.

### 2.1. Identifying candidate grains   

**Figure 2:**
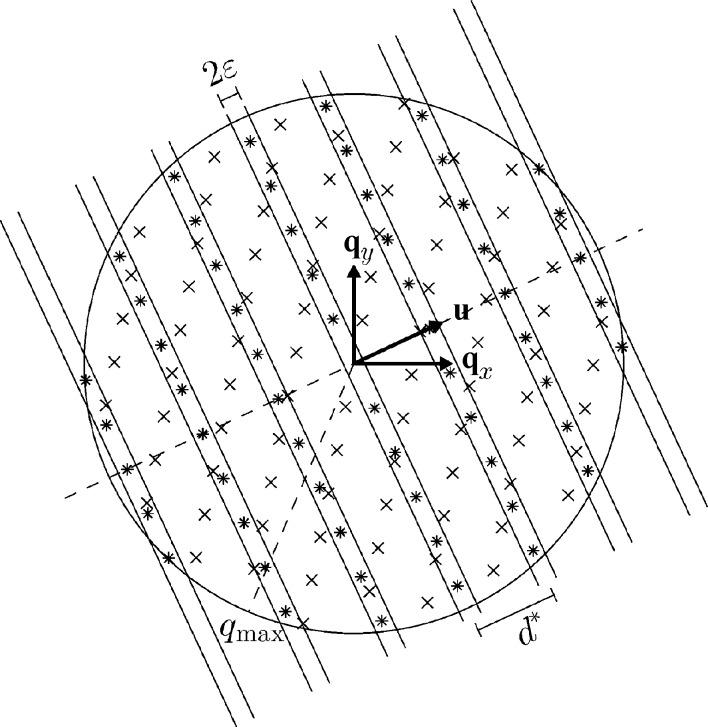
Illustration of the first step in the algorithm. For ease of visualization a two-dimensional slice through the center of reciprocal space is shown. We assume data are available up to a certain *q*_max_. The symbols * and × mark reflections from two grains. For a given direction 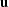, the lattice spacing d* is varied and the maximum number of reflections within the strips of fixed width 2∊ are counted.

**Figure 3:**
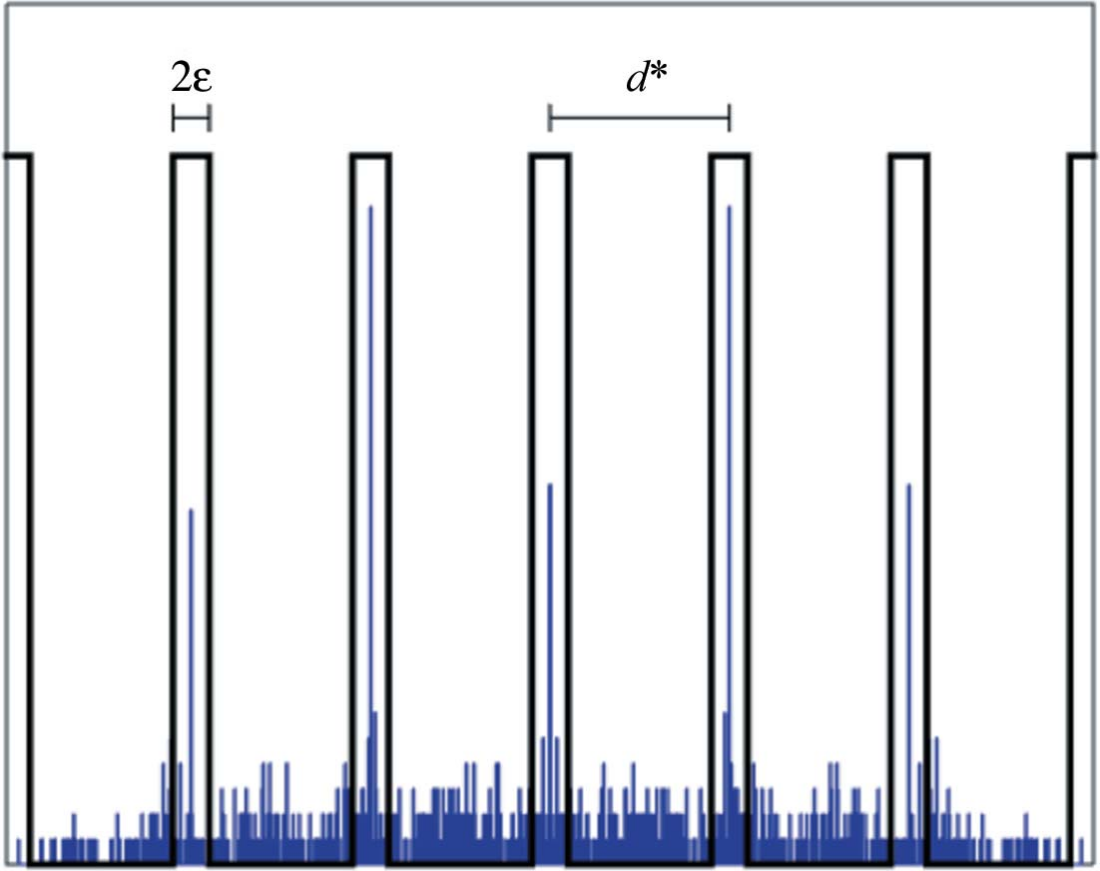
Illustration of the filter used to count reflections. The histogram is an example of the projection of the experimental data onto the line defined by 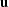 (*cf.* Fig. 2 ▸). The filter comprises a set of box functions of fixed width 2∊ and distance between box centers d*.

A number *N*_u1_ of directions 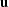 are randomly chosen in sample space (see Fig. 2 ▸). These are candidate lattice plane normals. For each 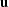 the reflections in the entire data set are projected onto the line defined by 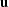. We then search for the one-dimensional lattice that fits most of these projected reflections. This is done by introducing a filter comprising a regular array of box functions (mathematically speaking a Dirac comb – also known as an impulse train function – convoluted with a box function; see Fig. 3 ▸). Let d* be the distance between the centers of the boxes, and 2∊ be the width of each box, with d* > ∊. ∊ is fixed and will typically be slightly larger than the experimental center-of-mass errors of the position of the reflections in sample space. At the expense of data analysis speed, this filter is used as it is more selective than a classical Fourier transform.

The parameter d*, the lattice plane spacing, is increased from a value d*_min_ (chosen to be smaller than *q*_min_) to d*_max_ = *q*_max_/2 in increasingly larger steps in such a way that the one-dimensional lattice point furthest from the origin (and closer than *q*_max_) only moves a distance of ∊ in each step, ensuring that no lattice points are missed by the counting. For each d*, we now count the number of reflections within the boxes and subtract a similar count rate for the case of the reflections being randomly placed in sample space. The optimal value of d* is defined as the value resulting in the highest number of counts.

Among the *N*_u1_ candidate lattice plane normals with corresponding optimal d* values, the ten candidates with the highest counts are kept for further investigation. For each candidate, the direction 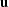 and the d* value are then further optimized by a local grid search, and these optimized values are used in the next step.

The step described above tends to produce subsets of the reflections that contain (almost) all of the reflections from one particular grain but also a significant number of reflections from other grains that by chance happen to be projected such that they fall within the boxes of the comb. In order to clean up these subsets, the above procedure is repeated on the subsets corresponding to the *N*_j_ best candidates: *N*_u2_ directions (not parallel to the original direction for that subset) are chosen randomly, and the best value of d* is found for each direction. Because of the smaller number of reflections the second search can be performed with a larger value of ∊ in order to speed up the algorithm. Out of these *N*_u2_ searches the ten best combinations (resulting in most counts) are then chosen, and the direction and value of d* are again optimized by a local grid search. After this the best candidate is saved. Since this is done for each of the *N*_j_ best candidates from the first step, this results in *N*_j_ subsets of reflections, each predominately originating from a single grain, and the task is now to identify the lattice basis vectors for each of these candidate grains.

### 2.2. Indexing of candidate grains   

For each of the *N*_j_ candidate grains found in step 1, a search is now performed for a (reciprocal) lattice basis. This is done by first listing all directions perpendicular to a plane spanned by three of the reflections (using only the 50 reflections closest to the origin, and not reflections with a cross product that is almost zero). The chosen subset of reflections is then as before projected onto each of these directions, and a search for the best one-dimensional lattice is performed. In this case the possible d* values are selected from the list of projected reflections (again between d*_min_ and d*_max_). The best value of d* is chosen as the largest d* value among those d* values corresponding to counts within 80% of the maximum count.

This results in a best d* value and the corresponding count from each of the searched directions, and from this set all d* values lower than 80% of the maximum count are removed. The remaining d* values and directions correspond to potential direct-lattice vectors pointing along the given direction and having length 1/d* (*e.g.* Giacovazzo *et al.*, 2011 ▸). From these potential direct-lattice vectors the three shortest, linearly independent candidates are found.

At this point the algorithm has produced a candidate unit cell (three real-space vectors) for *N*_j_ potential grains in the sample. From each of these the reciprocal lattice vectors are calculated, and for each reciprocal lattice point in this potential reciprocal lattice the nearest reflection (closer than 5∊) is found. This search for reflections in the potential lattice is performed in the full set of reflections. The number of reflections matching the potential lattice found in this way is then used to determine which of the *N*_j_ candidate unit cells is best by selecting the one corresponding to the most reflections, and if the number of reflections is high enough this candidate is then chosen for further optimization; if not, a new search is performed.

### 2.3. Optimization of the unit cell   

It is possible that the steps described above result in a potential unit cell that either is smaller or larger than the real unit cell of the grain or is given by a non-standard set of basis vectors. There are well known methods to determine a standard reduced unit cell (Gruber, 1973 ▸; Křivý & Gruber, 1976 ▸; Grosse-Kunstleve *et al.*, 2004 ▸). As cell reduction is not a main focus point here, we chose a simple heuristic solution.

By trying simple linear combinations of the found basis vectors, it is checked if a unit cell with a larger volume results in significantly (more than 20%) more reflections corresponding to the lattice. If this is the case this new unit cell is chosen over the original guess. Next, the orientation and length of the three potential direct lattice basis vectors are optimized, and the reflections corresponding to the found (candidate) grain are removed from the full set of reflections to obtain a new set of reflections which is then used as the full set of reflections, and the steps describes above are repeated. This continues until there have been a number *N*_t_ of consecutive tries where no potential unit cell with enough reflections has been found.

## 3. Simulations   

The aim of the simulation is to prove the concept and to provide an understanding of the accuracy and robustness of the algorithm.

The diffraction data that served as input to the simulations were generated by the program *PolyXSim* (Sørensen, 2006 ▸). The X-ray energy was defined to be 50 keV. Reflections were harvested and a normally distributed noise was added to each component of the position of each reflection.

Two studies were performed (each comprising six identical simulations): one with 500 grains of cementite, and one with 200 grains of four minerals typically found in granite (50 grains of each). The materials chosen reflect potential use for studies of minority phases in steel and for geological specimens, respectively. Furthermore, the minerals exhibit different crystal symmetries ranging from trigonal to monoclinic. The unit-cell parameters for the five materials are given in Table 1 ▸. All grains were assumed to be fully illuminated and of the same size, sufficiently large that signal-to-noise issues can be neglected. The parameters for the simulations are shown in Table 2 ▸. σ is the standard deviation of the noise added to each of the components of the position in reciprocal space of the various reflections.

As a measure of the success of the simulations a grain was defined as successfully identified if the unit-cell volume was within 1% of the nominal value and if the fraction of correctly identified reflections was above 0.9.

The results are shown in Table 3 ▸. For each material class (cementite and granite) the average results from ten simulations are presented. The table shows that almost all grains are successfully identified, with an accurate determination of the unit-cell volume, a very high level of average completeness and a very low level of falsely attributed reflections.

Notably, the figures of merit for the multiphase granite simulation in Table 3 ▸ are even better than for the single-phase cementite simulation. The data analysis speed was also much faster. This illustrates the fact that grains are indexed independently, and hence the figure of merit and running times are assumed to be approximately independent of the number of phases, but strongly dependent on the total number of grains.

It is also noteworthy that the program identified the grains of the four different phases in the granite simulation in the order of decreasing number of reflections per grain, that is plagioclase, orthoclase, biotite, quartz. This happens to be in order of decreasing unit-cell volume and increasing crystal symmetry.

## 4. Discussion   

### 4.1. Potential applications   

The output of the above program – the list of grains and associated reflections – can immediately be used in connection with a variety of existing single-crystal and multicrystal analysis programs. An overview of the status of (single-phase) multigrain crystallography was provided recently by Sørensen, Schmidt *et al.* (2012 ▸). Here examples are provided of resulting three-dimensional maps of grains, orientations and stresses in materials science and geoscience, as well as the use of multigrain structure solution and refinement in small-molecule drug design and photocatalysis studies. Included is also preliminary work on proteins. The fact that the algorithm presented above requires neither *a priori* information nor that a certain number of grains is present for each phase generalizes all of these applications to studies of multiphase materials with unknown space groups and an arbitrary number of grains of each phase.

In addition we point to the following prospective uses:

(*a*) Minority phases. Using powder diffraction it is often difficult to observe phases with a volume fraction smaller than 1%. In contrast, with multigrain methods, it is realistic to detect volume fractions of 10^−6^, provided the grains of the minority phase are sufficiently large to give rise to detectable diffraction spots (Poulsen *et al.*, 2001 ▸). Implemented with a suitable sample translation, it becomes possible to screen for diffraction signals from particles associated with parts per million concentrations.

(*b*) Local diffraction. With the upcoming synchrotron nano-beam beamlines it becomes possible to use scanning procedures to make three-dimensional maps of a set of nano-scale grains. As scanning methods tend to be slow, for larger samples it will be natural to map only the grains within an intrinsic gauge volume, which may be a small fraction of the total sample volume. The diffraction signal will in such cases be dominated by spurious diffraction spots moving in and out of the beam during the rotation. Analogous to the case of the *DIRAX* algorithm for use in single-crystal diffractometry with an obstinate list of reflections, the current algorithm is seen as being robust towards such outliers.

(*c*) Total crystallography. Sørensen, Schmidt *et al.* (2012 ▸) defined this concept as the simultaneous characterization of the three-dimensional atomic and the three-dimensional grain-scale structure of polycrystalline samples with unknown phase(s), as well as the temporal characteristics of such samples. In other words total crystallography is a (hypothetical) method that allows one to study an arbitrary polycrystal and for each grain characterize both its atomic structure and its mesocale structure (the three-dimensional shape, orientation and stress state). In our view the current work demonstrates the feasibility of total crystallography. Specifically, in a 3DXRD setup, far-field and near-field two-dimensional detectors can be used simultaneously (Poulsen, 2012 ▸; Hefferan *et al.*, 2012 ▸). It is therefore possible first to identify and index the grains using the far-field data and the method described here and then for each phase to use the near-field data and existing programs to map grains in three dimensions.

The generalization of the above approach to monochromatic or time-of-flight neutron multicrystal diffraction is seen as straightforward.

### 4.2. Limitations   

The indexing algorithm described here does not use any *a priori* information, and as such we anticipate that known and unknown phases will be handled equally well. Likewise, crystal symmetry is not used directly, and as such we anticipate that, everything else being equal, figures of merits will be independent of crystal symmetry. These hypotheses are corroborated by the simulations performed. Instead, applications will be restricted by the following inherent limitations (which apply to all multigrain indexing approaches):

(*a*) Spot overlap. The probability of spot overlap on the detector is determined by the number of grains illuminated, the texture of the sample, the size of the unit cell and the orientation spread of each grain. Experience from single-phase indexing methods shows that for typical samples of relevance to hard materials science one can aim at indexing up to a few thousand grains simultaneously, while for medium-sized crystal structures such as crambin, indexing of up to 100 grains is feasible (Sørensen, Schmidt *et al.*, 2012 ▸).

(*b*) Grain sizes. Typically grain volumes vary by several orders of magnitude. As a consequence, all reflections associated with large grains may be above the intensity threshold, all reflections for small grains may be below, while a fraction of the diffraction spots are identified for grains of intermediate size. For the latter grains an iterative approach may be needed.

An additional limitation of the current implementation is the speed of data processing. Indexing of the 500 cementite grains above took 5 d, while the 200 mineral grains took 1.5 d (using MATLAB and a single-core PC). We anticipate that the speed could be greatly increased by the use of a different programming language, GPUs and parallel computing, but still the approach may be too slow, for example, for on-line analysis of more challenging samples. There are a variety of ways to increase the speed, involving, for example, *a priori* information (some phases are known), sorting the data (searching first for the larger grains using only the more intense spots) or gaining additional information on grain position by acquiring data on an additional semi-transparent near-field detector. However, this is outside the scope of this article, where the aim has been to demonstrate the feasibility of multigrain indexing of multiphase samples.

## 5. Conclusion   

Conventionally, X-ray crystallography is based on two extreme sample morphologies: perfect single crystals and homogeneous powders. The multigrain methods developed over the past decade enable us to treat polycrystalline specimens as an ensemble of individual crystals, creating the possibility to rigorously characterize such samples in terms not just of average properties but of the distributions of those properties. The work in this article generalizes the previous work in the direction of multiphase materials. It also points to the feasibility of total crystallography: the synthesis of methodologies for three-dimensional grain mapping (mesoscale structure) and structure solution and refinement (atomic scale). More specifically, for a fixed number of grains and reflections the resulting figure of merit and efficiency of the algorithm presented do not depend on the number of phases. Likewise, the figure of merit and data processing speed are not strongly dependent on the group symmetry.

## Figures and Tables

**Table 1:** Unit-cell parameters for the materials used in the two types of simulations, of cementite and four minerals commonly found in granite

Name	Cementite	Quartz	Biotite	Orthoclase	Plagioclase
Crystal system	Orthorhombic	Trigonal	Monoclinic	Monoclinic	Triclinic
Space group	*Pnma*	*P*3_2_21	*C*2/*m*	*C*2/*m*	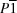
*a* (Å)	4.51	4.92	5.33	8.56	8.19
*b* (Å)	5.05	4.92	9.23	13.00	12.88
*c* (Å)	6.73	5.40	10.17	7.19	14.12
α (°)	90	90	90	90	93.30
β (°)	90	90	100.16	116.02	115.79
γ (°)	90	120	90	90	91.12
*V* (Å^3^)	153	113	493	719	1342

**Table 2:** Parameters used for the simulations

	Cementite	Granite
Number of grains	500	4 × 50
Number of reflections per grain	104	54, 130, 192, 702
∊ (Å^−1^)	0.0005	0.0005
*N*_u1_	10000	10000
*N*_u2_	5000	5000
*N*_j_	5	5
*q*_max_ (Å^−1^)	0.6	0.5
d*_min_ (Å^−1^)	0.1	0.05
*N*_t_	20	20
σ (Å^−1^)	0.0001	0.0001

**Table 3:** Results of the simulations Four figures of merit for the two materials classes defined in Table 1 ▸ are listed. The results represent the average of ten simulations with standard deviations in parentheses.

	Cementite	Granite
Fraction of grains successfully identified	0.9924 (4.7 × 10^−3^)	0.9985 (3.4 × 10^−3^)
Relative absolute deviation from correct unit-cell volume for successfully identified grains	1.5 × 10^−4^ (2.1 × 10^−4^)	9.4 × 10^−5^ (1.1 × 10^−4^)
Fraction of reflections correctly identified for each grain	0.9954 (4.4 × 10^−2^)	0.9989 (2.4 × 10^−2^)
Fraction of reflections incorrectly attributed to each grain	1.2 × 10^−3^ (7.3 × 10^−3^)	6.2 × 10^−5^ (7.8 × 10^−4^)
